# External decontamination of wild leeches with hypochloric acid

**DOI:** 10.1186/1471-2334-4-28

**Published:** 2004-08-25

**Authors:** Atakan Aydin, Hasan Nazik, Samet Vasfi Kuvat, Nezahat Gurler, Betigul Ongen, Serdar Tuncer, Emre Hocaoglu, Sinan Nur Kesim

**Affiliations:** 1 Department of Plastic and Reconstructive Surgery, Istanbul University, Istanbul Faculty of Medicine, Istanbul, Turkey; 2 Department of Clinical Microbiology, Istanbul University, Istanbul Faculty of Medicine, Istanbul, Turkey

## Abstract

**Background:**

Medicinal leech, *Hirudo medicinalis*, has been used in plastic and reconstructive surgery, to relieve venous congestion and to improve the microrevascularization of flaps. In many countries, wild leeches are still provided from local markets and utilised with antibiotic prophylaxies. In this research, results of identification of bacteria in the transport fluid is reported, oral and intestinal floras and the antibiograms of the identified microorganisms are investigated. Also, to avoid possible infections, the ability of hypochloric acid, a disinfectant, to suppress the relevant microorganisms without changing the life style and behavior of leeches in terms of sucking function, is investigated.

**Methods:**

Bacterial identifications and antibiograms of oral and intestinal flora and transport medium were performed for 10 leeches. The optimum concentration of hypochloric acid which eliminated microorganisms without affecting the viability and sucking function of the leeches were determined by dilution of hypochloric acid to 100, 50, 25, 12.5, 6.25 *ppm *concentrations in different groups of 25 leeches. Finally, 20 leeches were applied atraumatically to the bleeding areas of rats, the duration of suction was determined and compared statistically between the leeches treated and not treated with hypochloric acid solution.

**Results:**

*Aeromonas hydrophilia *was the most commonly identified microorganism and found to be resistant to first generation cephalosporins, frequently used in prophylaxis at surgical wards. In the next stages of the study, the leeches were subjected to a series of diluted hypochloric acid solutions. Although disinfection of the transport material and suppression of the oral flora of *hirudo medicinalis *were successful in 100, 50, 25, 12.5, 6.25 *ppm *concentrations; 12.5 *ppm *solution was the greatest concentration in which *hirudo medicinalis *could survive and sucking function was not affected significantly.

**Conclusions:**

External decontamination of wild leeches with 12.5 ppm hypochloric acid enables bacterial suppression without causing negative effects on leech sucking function and life.

## Background

The medicinal leech, *Hirudo medicinalis*, has been used with increasing frequency during the past few decades for salvage of venous compromised pedicled flaps, microvascular free-tissue transfers and replantations. Although the therapeutic use of leeches in medicine dates back 50 BC; for centuries they were collected from various water supplies and utilised under septic conditions with the risk of wound infection and infestation. The supply of leeches was modernized by medicinal leech farm set up in the 1970s [1].

**Figure 1:**
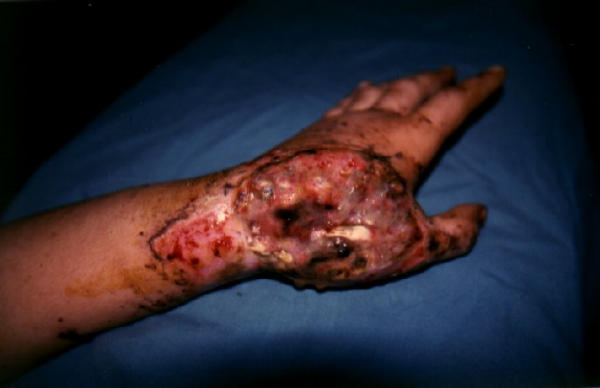
Tissue defect on the dorsum of the hand of a male patient after a crush type injury.

**Figure 2:**
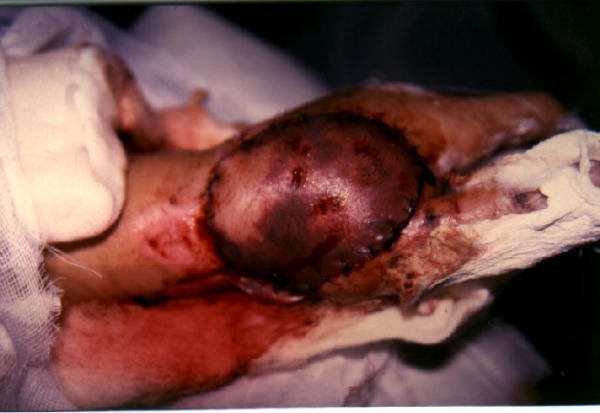
Venous insufficiency of the posterior interosseous flap used for defect reconstruction.

**Figure 3:**
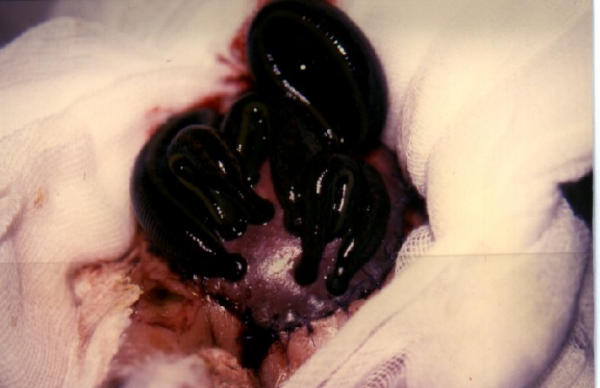
Leech usage in the treatment of venous insufficiency.

**Figure 4:**
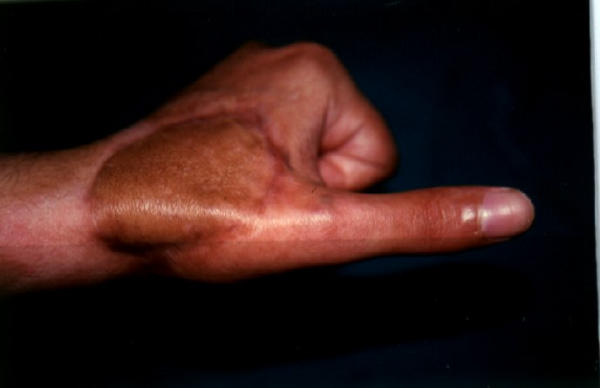
Late follow up of the salvaged flap.

Today, leech therapy is indicated in plastic and reconstructive surgery, to relieve venous congestion and to improve the microrevascularization of flaps (Fig 1,2,3,4) or replants with an increase in success rate by 60%–83%. However, in the presence of infection as a complication of medicinal use of leeches; the success rate for flap salvage may decrease to ≤ 30% [2]. *Aeromonas *is the most common microorganism in leech infections and may cause a wide spectrum of deseases such as cellulitis, ocular infections, arthritis, myocarditis, peritonitis, meningitis, bacteremia and sepsis [2-5]. In many countries, wild leeches are still provided from local markets and utilised in many plastic and reconstructive surgery clinics depending on antibiotic prophylaxies.

In the first stage of the study, bacterial content of the transport fluid of leeches that were bought from local markets were studied, also oral and intestinal flora cultures and antibiograms of the identified microorganisms were performed subsequently.

In the next stages hypochloric acid, a disinfectant, was tested on animals that were not bred in specific laboratories, to suppress the relevant microorganisms in order to avoid possible infections, without changing the life style and behavior of leeches in terms of sucking function.

## Methods

This study was approved by the Animal Care and Ethics Committee of our institution.

### Stage 1

**Figure 5:**
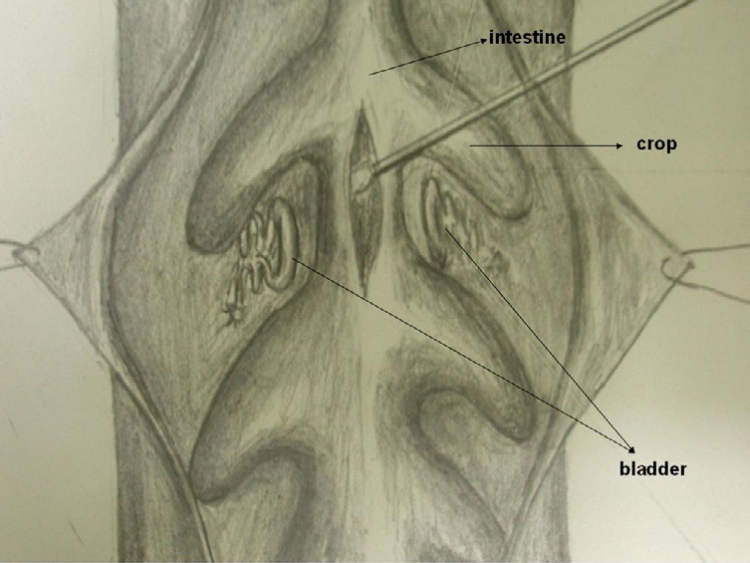
Intestinal exploration of Hirudo medicinalis and taking specimen with cotton swab.

Ten leeches were obtained with their 100 cc original water from different stores. Their sizes varied between 6–10 cm in length and 0, 5 – 1, 5 cm in width. They were transported in sterile boxes immersed in the water which the animals were kept at the market. For 2 days they were observed at room temperature. Specimens were taken and smears from transport fluids were prepared. All leeches were held with sterile gloves and prepared in sterile conditions. Specimens were obtained from their mouth (the smaller and more mobile pole) with the help of sterile cotton – swab. After traction was applied between the mouth and posterior muscular organ, the animal was cleaned with alcoholic solution of povidone-iodine, as described by Hokelek et al. [6]. A longitudinal incision was made on the animal and crop or intestine was exposed (Figure 5). Using sterile cotton-swabs, smears were prepared from the intestinal material and they were planted in MacConkey and blood agars in 5 minutes. The growth was observed after 24–48 hours of incubation at 37°C. In addition to classical bacterial identification methods, Analytical Profile Index 32 GN (BioMérieux, France) kit was also used for identification. Further biochemical studies were made on microorganisms that were found to be *A. hydro/caviae *and *A. sobria *for definite identification. Since bacterial growth was observed in specimens taken from oral and intestinal flora or transport fluid of all animals except one ; antibiograms for aeromonas isolates were performed.

### Stage2

30 leeches from different stores were obtained and transported in different sterile boxes with their 100 cc original water as in stage 1. On the following day, samples were taken from transport fluids and oral region and planted in MacConkey and blood agar. Twenty five leeches, which had bacterial growth in either oral flora or transport water were used for the second stage. 100, 50, 25, 12.5, 6.25 *ppm *solutions were prepared by adding apropriate amounts of hypochloric acid into the transport fluids of 25 leeches seperately with five animals in each concentration group. After 10 minutes of contact with hypochloric acid solutions at 20°C, smears were taken from mouths and transport fluids of each leech under sterile conditions with cotton-swabs. Cotton-swabs were put into a neutralizing media (lecithinized agar media) because they could also contain disinfectant fluid which would prevent microorganism reproduction. Then the neutralized smears were planted into MacConkey and blood agar [7]. The leeches were then put into boxes containing distilled water and observed. Although the leeches which had been left in the 100, 50, 25 *ppm *solutions lost their muscular activity and died ;the leeches which were treated with 12.5 and 6.25 *ppm *hypochloric acid solutions were alive. Bacterial growth was not observed in either concentration group for the specimens taken from oral flora and transport fluid, however bacterial cultures were positive for in specimens taken from the intestine or crop. Of these microorganisms, bacterial colonies for *Aeromonas spp *were counted. To compare the changes in the number of *Aeromonas *colonies in leech intestinal flora induced with the application of hypochloric acid, the intestine of five new leeches were exposed as described for Stage 1. Based on the findings of Stage 2, a concentration of 12.5 ppm was chosen for Stage 3 studies.

### Stage 3

20 leeches were obtained with their 100 cc original water from different stores and equally divided into two groups. One group was treated with hypochloric acid while the others had no treatment at all. After taking specimens from the transport fluids and oral floras, for 10 leeches 12.5 *ppm *hypochloric acid solution was prepared with the transport fluids. After waiting for 10 min at 20°C temperature, smears were taken from the oral floras and fluids again. All leeches were put into boxes containing distilled water and watched for survival and blood suction function on sedated rats (ketamine 30 mg/kg and xylazine 10 mg/kg). After shaving dorsal region of 20 rats, 2 mm long skin incissions were done in order to produce bleeding. The leeches were applied to the bleeding areas atraumatically and the duration of suction was determined and compared statistically (ANOVA) between the leeches treated and not treated with hypochloric acid solution. Statistical significance was presumed at *p *< 0.05.

## Results

### Stage 1

Bacterial growth was observed in specimens taken from the oral flora in 4 animals (40%), intestinal flora in 9 animals (90%) and transport media in 5 animals (50%). In one leech, no growth was observed in either oral, intestinal and transport fluid cultures. In all culture media, 4 different gram (-) bacterial growth (*aeromonas species*, *peudomonas species*, *acinetobacter species*, *sphingobacterium species*) was observed. In one media, Gram (+) growth was seen :*meticilline susceptible coagulase (-) staphylococcus*. Dominant microorganisms were *aeromonas species*, Table 1. This was an expected result. The biochemical characterization and identification of *Aeromonas spp*. was investigated by using conventional methods and a commercial kit ID 32 GN ATB System (BioMérieux). In this study, with this system the results were obtained as *A. hydro/caviae*, *A. sobria*, *A. salmonicidia and A. spp*, with the latter nomenclature designating all other *Aeromonas species*. The most commonly encountered agents, *A. hydro/caviae *and *A. sobria *underwent further biochemical studies, as suggested by Abbott, et al.[4]. As a result, it was seen that *A. hydro/caviae *was in fact *A. hydrophila*, and *A. sobria *was in fact *A. veronii biovar sobria *(Table 2) Antimicrobial susceptibility testing was performed by the disc diffusion method according to NCCLS (National Commity Clinical Laboratory Standart / American). The *Aeromonas spp*. were found to be susceptible to third generation cephalosporines, aminoglycosides and co-trimoxazole, and all aeromonas spp. were resistant to ampicillin-sulbactam and amoxycillin-clavulanic acid (Table 3). Three *Aeoromonas spp*. were found to have inducable beta lactamase which meant that although they were susceptible, they might develop resistance to beta lactams other than carbapenems during treatment.

### Stage 2

Fluid and oral flora cultures that were prepared before hypochloric acid application showed multiple microorganism growth, as it was in stage 1. After adding hypochloric acid there was no growth in transport fluid and oral media cultures in either concentration so that no identification and antibiogram study could be performed. After 10 minutes in hypochloric acid solution, leeches were taken to sterile containers which contained distilled water. The muscle activity of all leeches that had been treated in 100 *ppm *solution ended in 3 min, in 50 *ppm *ended in 7 min, in 25 *ppm *ended in 21 min and they died eventually. The ones which had been immersed in 12.5 *ppm *and 6.25 *ppm *solutions survived. The *Aeromonas *colonies in the intestinal flora of these last two groups were compared with the control animals'. With respect to colony suppression, while the difference between the control group, 12.5 ppm group was significant, (p < 0.05, F = 30.1, ANOVA) however the difference between control group and 6.25 ppm group was not significant.

### Stage 3

All the leeches survived while hyperactivity followed by hypoactivity was seen in the group of leeches treated with hypochloric acid. In this group, two of the leeches never made suction but the other eight leeches sucked the bleeding area for 5–16 min (mean 12.2 ± 3.8). Non hypochloric acid treated group sucked for 9–22 min (mean 15.5 ± 4.4) and the difference between two groups was not significant (p > 0.05, F = 2.6, ANOVA).

In 70 % of cultures that were prepared from transport fluids of leeches and in 50% of cultures that were prepared from oral floras of leeches before hypochloric acid application, bacterial growth (mainly *Aeromonas spp*) were observed. After hypochloric acid application no growth was observed in any of the specimens taken from animals, therefore no identifications or antibiograms could be performed.

## Discussion

Many reports showed that venous compromise is a more common complication than arterial occlusion in free-tissue transfers. Although surgical correction is the first choice for management of venous compromised flaps ; in cases where surgical correction is not feasible or fails, nonsurgical procedures such as exanguination treatments can be used. Among them, medicinal leech therapy has become widely accepted to promote perfusion of venous compromised flaps and to relieve congestion. However there are reports in the literature concerning about infections specially with aeromonas bacteria after leech therapy calling attention for appropriate antibiotic prophylaxis [8,9].

Many species of *Aeromonas *survive in water. These also can live in materials embedded in water, drainage tubes, fountains and containers for distilled water. Among other aeromonas species *A. hydrophilia *is the mostly isolated one from the intestines of leeches. Hemoglobin that is sucked by the leech is denaturated by *aeromonas*. One of the products, heme, is used by aeromonas and the other, globulin, is used by the leech[10]. This shows the endosymbiotic relationship of leeches and aeromonas. Many investigators isolated *aeromonas spp *as the dominant bacteria in infections related with leech usage [11-13]. *Aeromonas spp*. can cause infections with contamination through the bite point of the leech or on the macerated skin. The infections caused by these microorganisms can be minimized by easy measures. Leeches should not be handled with the forceps from the containers because these traumas can cause regurgitation of the leech into the wound and eventual contamination. Therefore, leeches should be taken with sterilized gloves without traumatisation of the animal. Same care should be taken while carrying the leech from the wound. When the leech is filled up, it puts itself off. If it is taken before this time, the risk of regurgitation is high. Low amounts of cigarette smoke or a heat source can be used in order to cause the leech to put itself off. During leech application, the routine surgical antibiotic prophylaxis must be changed because aeromonas spp. are resistant to first generation cephalosporins which are widely used at surgical wards. These are usually susceptible to third generation cephalosporins, aminoglycosides, tetracyclines and quinolones. Lineaweaver et al. recommended third generation cephalosporins for the prophylaxis of surgical procedures after which leeches were utilised [10,12].

In countries, where special farms for medicinal leeches do not exist, animals obtained from markets should be studied for their floras and antibiotic susceptibilities. Eroğlu et al. investigated the floras and the antibiotic susceptibility of leeches in Black Sea region. Most commonly *A. hydrophila*, *Ochrobacter antropia*, *nonfermantating gram (-) rods*, *Acinetobacter Iwofii *and *A sobria *were isolated. All of the isolates were sensitive to ciprofloxacine, cefotaxime, ceftazidime, gentamycine and trimetoprim-sulfamethoxasole [14]. Likewise, we aimed to investigate the general flora of leeches we use occasionally and antibiotic susceptibility of these bacteria. Our results correlated with the other studies about this subject in the literature. The interesting observation in our study was there were 3 *Aeromonas hydrophylia *colonies which produced inducable betalactamase. In case of infections with these species, beta lactam antibiotics except carbapenems should not be used.

In addition, we aimed to supress possible bacterial contamination of the transport media and oral floras of leeches but not intestinal flora because of the endosymbiotic relationship of the aeromonas and leech, with hypochloric acid solutions at disinfectant concentrations that does not affect the life style of leeches.

Investigators have attempted to disinfect the guts of leeches before they are placed on patients by placing the ectoparasites in % 0.02 chlorhexidine for 15 seconds or in antibiotic solutions (tetracycline or cefoperazone solutions) for 12 hours, but these attempts were unsuccessful [2]. Mackay et al. incubated leeches in solutions of antibiotics to which *Aeromonas spp*. are sensitive for 12 hours but could not eradicate *aeromonas *from the intestines [11].

We preferred hypochloric acid which is an ideal disinfectant and has a wide antibacterial spectrum for desired purpose. Chlorine in the form of hypochloric acid exhibits rapid microbicidal activity by inhibiting of key enzymatic reactions within the cell and eventual protein denaturation. It has a rapid bactericidal effect and can dissolve in water. However, it has some disadvantages as it irritates mucosal membranes, has interactions with some chemicals and metals [15].

It was showed that bactericidal effect of hypochloric acid of 100 *ppm *concentrations on pseudomonas was achieved within 10 min exposure [7]. *Aeromonas spp*. cause similar nosocomial infections and has similar antibiotic susceptibility with *pseudomonas spp*. Therefore, in the second stage of our study, beginning with 100 *ppm*, we applied decreasing concentrations of hypochloric acid in transportation fluids, observed bacterial growth by taking specimens from transport material and oral flora of *hirudo medicinalis*. We tried to find the dilution ratio that was closest to 100 *ppm*, suppresing oral flora and transfer liquid and also allowing the leech to survive with normal function. As a result of the second stage of the study we found out that concentration to be 12.5 *ppm*.

In the third stage, we observed that mean sucking duration of the hypochloric acid treated group was shorter than the other group but the difference was not significant (12.2 min & 15.5 min respectively). However hypochloric acid had successful disinfection effect for transport liquid and oral flora. In our opinion, such a decrease in duration of sucking function can be preferable to infection possibility. Since leeches are much cheaper than antibiotics, it is logical to use them once, after treating with hypochloric acid, and never utilise them again.

We do not advise to use "full-up" leeches over and over again by putting them in hypertonic solutions to force them vomitting because if they regurgitate, the intestinal flora can readly contaminate the environment where the leeches are kept and cause infection eventually. Although the oral flora and transport enviroment studies do not really address the problem since excretory contamination is probably the major factor in leech infections ; we believe that to take any measure in order to decrease the infection risk during utilisation of ordinary leeches is valuable.

## Conclusions

We can comment that preparation of 12.5 *ppm *hypochloric acid solution with transport fluids of ordinary leeches obtained from the local market for 10 minutes and then taking the leeches gently from water before application can prevent possible infections caused by contamination from leech oral flora and transport medium.

## Competing interest

None declared.

## Author's contributions

AA and SVK participated in design of the study, involved in the dissections of leeches. EH and ST participated in the design of the study and writing of the manuscript.HN, NG and BO involved in the antibiogram tests. SNK participated in design of the study.

## Pre-publication history

The pre-publication history for this paper can be accessed here:



## Figures and Tables

**Table 1:** Results of the cultures prepared from transport fluids, oral and intestinal smears of 10 leeches.

**Number of leech**	**Source of the culture material**	**Bacterial Growth**
**1**	oral flora	no reproduction
	intestinal flora	no reproduction
	transport fluid	no reproduction
**2**	oral flora	Aeromonas hydro/cavie, Aeromonas spp, Acinetobacter spp
	intestinal flora	Aeromonas hydro/cavie, Aeromonas spp, Acinetobacter spp
	transport fluid	Aeromonas hydro/cavie, Aeromonas spp, Acinetobacter spp
**3**	oral flora	no reproduction
	intestinal flora	Aeromonas hydro/cavie
	transport fluid	no reproduction
**4**	oral flora	no reproduction
	intestinal flora	Aeromonas hydro/cavie
	transport fluid	Aeromonas hydro/cavie, Aeromonas spp, Acinetobacter spp
**5**	oral flora	no reproduction
	intestinal flora	Aeromonas sobria
	transport fluid	no reproduction
**6**	oral flora	no reproduction
	intestinal flora	Aeromonas sobria
	transport fluid	Acinetobacter spp, methicillin sensitive-coagulase negative Staphylococcus spp
**7**	oral flora	Aeromonas salmonicidia, Pseudomonas spp, Sphingobacterium spp
	intestinal flora	Aeromonas hydro/cavie, Aeromonas sobria
	transport fluid	Aeromonas hydro/cavie, Aeromonas salmonicidia
**8**	oral flora	Aeromonas hydro/cavie
	intestinal flora	Aeromonas sobria
	transport fluid	Pseudomonas spp
**9**	oral flora	Aeromonas hydro/cavie
	intestinal flora	Aeromonas hydro/cavie
	transport fluid	no reproduction
**10**	oral flora	no reproduction
	intestinal flora	Aeromonas hydro/cavie
	transport fluid	no reproduction

**Table 2:** Definitive identification of A. hydro/caviae and A. sobria with biochemical tests.

**No**	**2**			**3**	**4**		**5**	**6**	**7**			**8**		**9**		**10**
**◆**	**O**	**I**	**F**	**I**	**I**	**F**	**I**	**I**	**I**		**F**	**O**	**I**	**O**	**I**	**I**

**Results of API 32 GN**	h/c	h/c	h/c	h/c	h/c	h/c	s	s	h/c	s	h/c	h/c	s	h/c	h/c	h/c
**M**	+	+	+	+	+	+	+	+	+	+	+	+	+	+	+	+
**Esculin**	+	+	+	+	+	+	-	-	+	-	+	+	-	+	+	+
**Catalase**	+	+	+	+	+	+	+	+	+	+	+	+	+	+	+	+
**Indole**	+	+	+	+	+	+	+	+	+	+	+	+	+	+	+	+
**VP**	+	+	+	+	+	+	+	-	+	+	+	+	+	+	+	+
**LDC**	+	+	+	+	+	+	+	+	+	+	+	+	+	+	+	+
**ODC**	-	-	-	-	-	-	-	-	-	-	-	-	-	-	-	-
**DNase**	+	+	+	+	+	+	+	+	+	+	+	+	+	+	+	+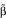 **Hem.**	+	+	+	+	+	+	+	+	+	+	+	+	+	+	+	+
**Glucose**	+	+	+	+	+	+	+	+	+	+	+	+	+	+	+	+
**Lactose**	-	-	-	-	-	-	-	-	-	-	-	-	-	-	-	-
**Urea**	-	-	-	-	-	-	-	-	-	-	-	-	-	-	-	-
**Citrate**	+	+	+	-	+	+	-	-	-	+	-	+	+	+	+	-
**Results**	h	h	h	h	h	h	v	v	h	v	h	h	v	h	h	h

**Abbreviations : **No: Number of leech, ◆: Source of the culture material, API 32 GN: Analitycal Profile Index 32 GN (BioMérieux), O: Oral flora, I: Intestinal flora, F: Transport fluid, h/c: A. hydro/caviae, h: A. hydrophila, s: A. sobria, v: A. veronii biogroup sobria, M: Motility, VP: Voges-Proskauer, LDC: Lysine decarboxylase, ODC: Ornithine decarboxylase, 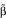
Hem: 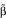
Hemolysis, Lactose: Asit from Lactose.

**Table 3:** The antibiograms of aeromonas isolates from 9 different leeches.

	**Leech**								
**Antibiotics**	**1st**	**2nd**	**3rd**	**4th**	**5th**	**6th**	**7th**	**8th**	**9th**
	h	h	h	v	v	h	h	h	h
**AMP**	R	R	R	R	R	R	R	R	R
**SAM**	R	R	R	R	R	R	R	R	R
**AMC**	R	R	R	R	R	R	R	R	R
**CZ**	R	R	R	S	S	R	R	R	S
**CXM**	S	S	S	S	S	S	S	S	S
**FOX**	S	S	S	S	S	S	S	S	S
**CRO**	S	S	S	S	S	S	S	S	S
**SCF**	S	S	S	S	S	S	S	S	S
**FEP**	S	S	S	S	S	S	S	S	S
**GM**	S	S	S	S	S	S	S	S	S
**TOB**	S	S	S	S	S	S	S	S	S
**NET**	S	S	S	S	S	S	S	S	S
**AK**	S	S	S	S	S	S	S	S	S
**SXT**	S	S	S	S	S	S	S	S	S
**NOR**	S	S	S	S	S	S	S	S	S
**IPM**	S	S	S	S	S	S	S	S	S
**MEM**	S	S	S	S	S	S	S	S	S
**TZP**	S	S	S	S	S	S	S	S	S
**AZM**	S	S	S	S	S	S	S	S	S
**IBL**		+					+	+	

**Abbreviations : **h:A. hydrophila, v:A. veronii biogroup sobria, R:Resistant, S:Sensitive AMP:Ampicillin, SAM:Sulbactam/ampicillin, AMC:Amoxicillin/clavulanate, CZ:Cefazolin, CXM:Cefuroxim FOX:Cefoxitin, CRO:Ceftriaxone, SCF:Sulbactam/cefoperazone, FEP:Cefepime, GM:Gentamicin, TOB:Tobramycin, NET:Netilmicin, AK:Amikacin, SXT:Trimethoprim/sulfamethoxazole, NOR:Norfloxasin, IPM:Imipenem, MEM:Meropenem, TZP:Piperacillin/tazobactam, AZM:Aztreonam,

**Table 4:** The number of bacterial colonies before and after hypochloric acid applications of 12.5 *ppm *and 6.25 *ppm *concentrations and control group.

**No**	***Concentration of hypochloric acid***	**Transport fluid Colony count**	**Mouth Colony count**	**Gut Colony count**
**1**	**before**	12	16	Not studied
	***12.5 ppm *after**	No reproduction	No reproduction	25
**2**	**before**	23	14	Not studied
	***12.5 ppm *after**	No reproduction	No reproduction	18
**3**	**before**	30	34	Not studied
	***12.5 ppm *after**	No reproduction	No reproduction	50
**4**	**before**	25	16	Not studied
	***12.5 ppm *after**	No reproduction	No reproduction	44
**5**	**before**	18	No reproduction	Not studied
	***12.5 ppm *after**	No reproduction	No reproduction	30
**1**	**before**	No reproduction	20	Not studied
	***6.25 ppm *after**	No reproduction	No reproduction	100
**2**	**before**	27	7	Not studied
	***6.25 ppm *after**	No reproduction	No reproduction	120
**3**	**before**	20	No reproduction	Not studied
	***6.25 ppm *after**	No reproduction	No reproduction	72
**4**	**before**	No reproduction	8	Not studied
	***6.25 ppm *after**	No reproduction	No reproduction	95
**5**	**before**	15	12	Not studied
	***6.25 ppm *after**	No reproduction	No reproduction	75
**1**	**Control group**	Not studied	Not studied	100
**2**		Not studied	Not studied	150
**3**		Not studied	Not studied	120
**4**		Not studied	Not studied	105
**5**		Not studied	Not studied	140
